# Disseminated Cryptococcosis in an Immunocompetent Patient: A Case Report

**DOI:** 10.1155/2012/652351

**Published:** 2012-04-18

**Authors:** S. Suchitha, C. S. Sheeladevi, R. Sunila, G. V. Manjunath

**Affiliations:** Department of Pathology, JSS Medical College, JSS University, Mysore 570022, India

## Abstract

*Cryptococcus neoformans* is ubiquitous encapsulated yeast found throughout the world. It predominantly causes significant infections in immunocompromised individuals, of which 80–90% occur in people with human immunodeficiency virus (HIV) infection. Disseminated cryptococcal infection is uncommon and almost always occurs in HIV-infected patients. Disseminated disease, especially noncutaneous cryptococcal abscess in immunocompetent hosts, is exceedingly rare. We report a case of disseminated cryptococcosis with soft tissue, pulmonary, and cerebral involvement in an otherwise healthy immunocompetent patient initially diagnosed by fine needle aspiration cytology (FNAC).

## 1. Introduction

*Cryptococcus neoformans* is opportunistic yeast commonly found in soil contaminated by bird feces throughout the world [1]. Cryptococcosis continues to cause significant morbidity and mortality in immunocompromised as well as immunocompetent patients [2]. Noncutaneous cryptococcal abscess without osseous involvement in an immunocompetent host is exceedingly rare, and, to our knowledge, soft-tissue cryptococcal abscess in an immunocompetent host has been previously reported only once in the literature [1].

## 2. Case Report

**Figure 1:**
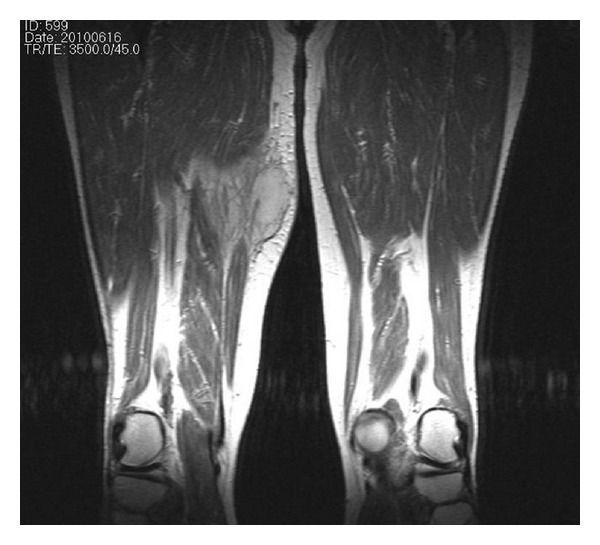
T2-weighted sagittal MRI of the thigh showing a heterogenous mass in the medial aspect of the right thigh.

**Figure 2:**
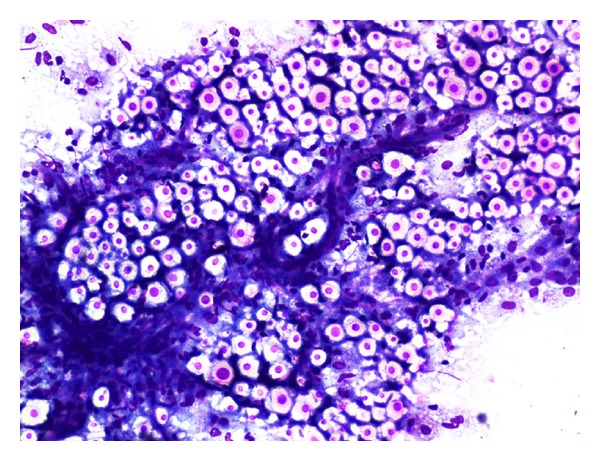
Numerous yeast cells surrounded by halos with endothelial cells and epithelioid cells (MGG, 100×).

**Figure 3:**
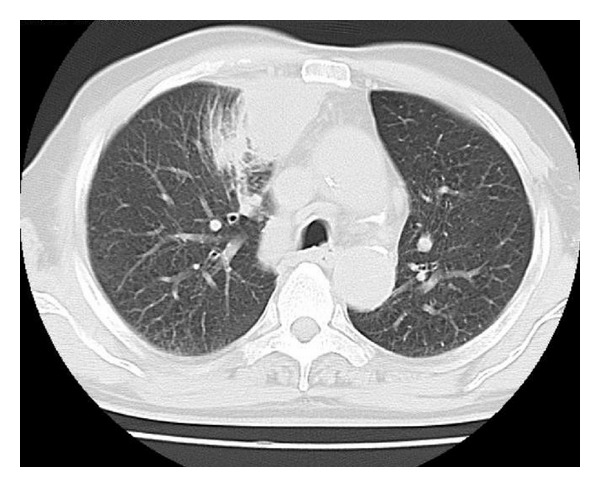
Spiral CT of the thorax showing an irregular mass lesion in the anterior and superior mediastinum.

A 70-year-old male patient presented with a solitary slowly increasing painless swelling in the medial aspect of the right thigh of one-and-a-half-month duration. The swelling measured 5.5 × 4 cm was firm in consistency, and the skin over the swelling was shiny. A clinical diagnosis of soft tissue sarcoma was made. A magnetic resonance imaging (MRI) revealed a heterogeneous mass of 6.5 × 5.2 × 4.5 cm in the medial compartment of the right thigh, in relation to the adductor longus and the vastus medialis muscles, in the inter- and intramuscular planes and extending to subcutaneous tissue (Figure 1). The clinical and radiological diagnosis was that of a soft tissue sarcoma. There was no history of use of immune-modulating medications. He was not a known diabetic. Hematological investigations were within normal limits except for erythrocyte sedimentation rate (ESR), which was 70 mm at the end of one hour (Westergren's method). Random blood sugar was 102 mg/dL. The patient was nonreactive for HIV1 and 2 by fourth-generation ELISA. FNAC was done. The aspirate was mucoid. It revealed the presence of budding yeast cells surrounded by halos with epithelioid cells, macrophages and endothelial cells in a mucoid background (Figure 2). Special stains, periodic acid Schiff (PAS) and mucicarmine with culture of the aspirated material, identified the organisms as Cryptococcus neoformans var *gattii*. Retrospectively, the patient was investigated for pulmonary and cerebral involvement. Chest X-ray showed right-sided hilar opacities, and spiral computerised tomography (CT) of the thorax showed an irregular mass lesion measuring about 6 cm in the anterior and superior mediastinum (Figure 3). Sputum culture showed the presence of cryptococcal organisms. MRI of the brain revealed a well-defined nodule of 1.72 × 1.44 cm in the posterior aspect of the right parietooccipital lobe in its gray-white matter junction, hyperintense on T2 and FLAIR sequences with hypointense rim. Perilesional edema was observed and was consistent with a diagnosis of cryptococcoma. Blood culture was negative for cryptococcal organisms. CD4+ T cell count was normal.

The patient was started on oral fluconazole and intravenous amphotericin B. No surgical debridement was done. On follow up at one year, he is clinically asymptomatic and radiological resolutions of his lesions are seen.

## 3. Discussion

 Cryptococcus neoformans has a complex polysaccharide capsule with antiphagocytic properties. Low concentrations of anticryptococcal antibodies (typically immunoglobulin M) are normally found in immunocompetent people because of daily exposure [3]. Although exposure in some regions of the world is nearly ubiquitous, this organism rarely causes clinically important infection in immunocompetent hosts. In contrast, it has become a notable opportunistic infection in those possessing a compromised cell-mediated immune response [4]. *Cryptococcus neoformans* var. *neoformans* (*C. neoformans*) is the species predominantly reported from immunocompromised patients, while *Cryptococcus neoformans* var. *gattii* (C. *gattii*) infection has been associated with immunocompetent patients [5].

Disseminated cryptococcosis is defined by (1) a positive culture from at least two different sites or (2) a positive blood culture [6]. In the present case, positive cultures were obtained from two sites.

Cases of cryptococcal infections in an immunocompetent host have been reported and primarily include pulmonary manifestations and cutaneous lesions [5, 7]. A history of a hobby or occupational exposure to soil, dust, sticks, or bird feces was noted in most cases [8]. However, no such history was available in the present case. It is important to recognize, however, that untreated cryptococcal infection has resulted in severe pulmonary infections with respiratory failure, or systemic dissemination even in immunocompetent hosts [7].

The radiographic and clinical presentation in the present case was that of a soft tissue sarcoma. FNAC helped in establishing an early diagnosis and also obtain material for culture.

Cryptococcosis is a sentinel infection that signals the perturbation of the host immune status [3]. Treatment of systemic manifestations of cryptococcal infections remains debated and nonstandardized [1]. In a patient reported in the literature, surgical debridement in conjunction with twelve weeks of oral fluconazole was successful in treating this soft tissue abscess [1]. However, in the present case, the patient responded to oral fluconazole and intravenous amphotericin B without surgical debridement.

 FNAC can sometimes be much more than an initial cytological investigation and can help uncover unsuspected and unlikely diagnosis. The initial diagnosis and confirmation of the similar nature of disseminated lesions helped in the early institution of specific antimicrobial therapy, even before the patient developed symptoms of the disseminated disease.

## References

[1] Gaskill T, Payne D, Brigman B (2010). Cryptococcal abscess imitating a soft-tissue sarcoma in an immunocompetent host: a case report. *Journal of Bone and Joint Surgery Series A*.

[2] Lui G, Lee N, Ip M (2006). Cryptococcosis in apparently immunocompetent patients. *QJM*.

[3] Matsushita T, Suzuki K (1985). Spastic paraparesis due to cryptococcal osteomyelitis: a case report. *Clinical Orthopaedics and Related Research*.

[4] Revenga F, Paricio JF, Merino FJ (2002). Primary cutaneous cryptococcosis in an immunocompetent host: case report and review of the literature. *Dermatology*.

[5] Capoor M, Nair D, Deb M, Gupta B, Aggarwal P (2007). Clinical and mycological profile of cryptococcosis in a tertiary care hospital. *Indian Journal of Medical Microbiology*.

[6] Yehia BR, Eberlein M, Sisson SD, Hager DN (2009). Disseminated cryptococcosis with meningitis, peritonitis, and cryptococcemia in a HIV-negative patient with cirrhosis: a case report. *Cases Journal*.

[7] Núñez M, Peacock JE, Chin R (2000). Pulmonary cryptococcosis in the immunocompetent host: therapy with oral fluconazole—a report of four cases and a review of the literature. *Chest*.

[8] Christianson JC, Engber W, Andes D (2003). Primary cutaneous cryptococcosis in immunocompetent and immunocompromised hosts. *Medical Mycology*.

